# Approach to ovarian torsion with corpus luteum re-moval in early pregnancy

**DOI:** 10.5339/qmj.2023.22

**Published:** 2023-12-12

**Authors:** Mona A. Hatata, Dalia A. Soliman, Khadija A. Hosni

**Affiliations:** ^1^Gynecology & Obstetrics Department, Al-Ahli Hospital, Doha-Qatar Email: hatatam@ahlihospital.com ORCID iD: 0000-0001-6435-0745

**Keywords:** Corpus luteum, First trimester, Progesterone, Ovarian torsion

## Abstract

The ovary’s Torsion is the adnexa’s total or partial rotation around its vascular axis or pedicle. Complete torsion causes venous and lymphatic blockade leading to stasis, venous congestion, hemorrhage, and necrosis. If neglected, it may result in permanent loss of the affected ovary. Ovarian torsion is one of the most critical gynecological surgical emergencies. Therefore, it requires urgent surgical intervention. It is mainly diagnosed by clinical examination and ultrasound. Ovarian torsion is primarily seen in the first trimester of pregnancy.^1,2^ Infertility treatment is a significant risk factor due to ovarian stimulation. We present a case of acute abdominal pain who came to the emergency department of Al Ahli Hospital, diagnosed as right adnexal torsion with viable intrauterine pregnancy at six weeks. Laparoscopic oophorectomy was done by removing the corpus luteum cyst, which supports early pregnancy. Despite its disposal, the pregnancy continued because the hormonal therapy started in doses higher than recommended for threatened abortion.^3^ Eventually, she gave birth to a healthy baby boy at 35 weeks.

## Introduction

Torsion of the ovary, tube, or both is responsible for about 2.7-7.4 % of all gynecological emergencies.^4^ The calculated incidence of ovarian torsion in pregnancy is 1-5 in 10,000 pregnancies.^5^ Ovarian torsion is frequently increasing due to the growing prevalence of ovarian stimulation treatment, which may cause multiple large ovarian follicular cysts.^6^ Ovarian hyperstimulation occurs more commonly with gonadotrophin injection than with clomiphene citrate.^7,8^

The right ovary is more likely to undergo torsion compared to the left. This could be due to the right utero-ovarian ligament being longer than the left or the presence of the sigmoid colon in the left pelvis that may limit the movement of the ovary and prevent torsion.^2^

Adnexal masses are commonly identified in pregnancy, with a 0.19-8.8% prevalence.^9^ The most common cause of adnexal masses during pregnancy is the corpus luteum cyst, which usually regresses spontaneously by the second trimester when the placenta takes over the progesterone production.^10^ Other clinically significant adnexal masses in pregnancy are hemorrhagic cysts, follicular cysts, benign teratomas, ovarian fibromas, and the serous cystadenomas.^11^

Usually, adnexal torsion presents with acute abdominal and pelvic pain of crescendo type, nausea, vomiting, abdominal tenderness, and signs of peritoneal irritation. Clinical examination may reveal a tender mass separated from the uterus.^12^ Ultrasound examination usually shows a tender, enlarged, edematous adnexal mass, often with a vascular center.^13^ The twisted vascular pedicle may be seen as a “whirlpool” sign. The presence of flow within the ovary does not exclude the diagnosis of ovarian torsion. Complete loss of Doppler signals within the ovary is a late sign.^14^

There is increasing evidence that MRI can be used in pregnancy for diagnosing and evaluating adnexal masses in pregnancy in cases where the ultrasound does not conclude the diagnosis.^15^ Moreover, removing the corpus luteum cyst during pregnancy may lead to a miscarriage due to progesterone production failure. Therefore, pregnancy will not be supported. Adequate progesterone replacement, in this case, is crucial to help the pregnancy to continue.^16^ It is worth noting that we found no previous similar cases in the literature presenting the removal of the corpus luteum in early pregnancy at six weeks.

## Case Report

This case concerns a 33-year-old woman with second gravida para 0, with previous first-trimester abortion. The patient did not report any significant past surgical or medical history except subfertility in the last five years. She presented to the emergency department (ED) at Al-Ahli Hospital on the 22^nd^ of March 2017 with a missed period; her last menstrual period (LMP) was on the 6^th^ of February 2017. Her chief complaint was acute lower abdominal and pelvic pain on the right side radiating to the groin and back, sharp in character and of sudden onset, with no history of vaginal bleeding or discharge. The pain was partially relieved by analgesics. She also had repeated vomiting and loose stool. She stated that her symptoms started the previous day. She did not mention any history of fever, urinary symptoms, or recent illness. She received Clomid (Clomiphene citrate, Sanofi Aventis) and Choriomon 5000 IU (Human chorionic gonadotrophin, IBSA) in her last cycle for ovulation induction. She had no check-ups until she presented to the ED with pain.

On examination, the patient was conscious, with severe generalized abdominal tenderness emphasized more on the right side, with rebound tenderness on the right iliac fossa. A vaginal examination showed a closed cervix with no vaginal bleeding. A bimanual examination revealed fullness in the right iliac fossa with severe tenderness. Vital signs were as follows: blood pressure: 128/72 mmHg; heart rate: 81bpm; temperature: 37.7˚C; O2 saturation: 100%; and respiratory rate: 20 breaths/min. Cardiovascular and respiratory systems were normal on examination.

**Figure 1.**
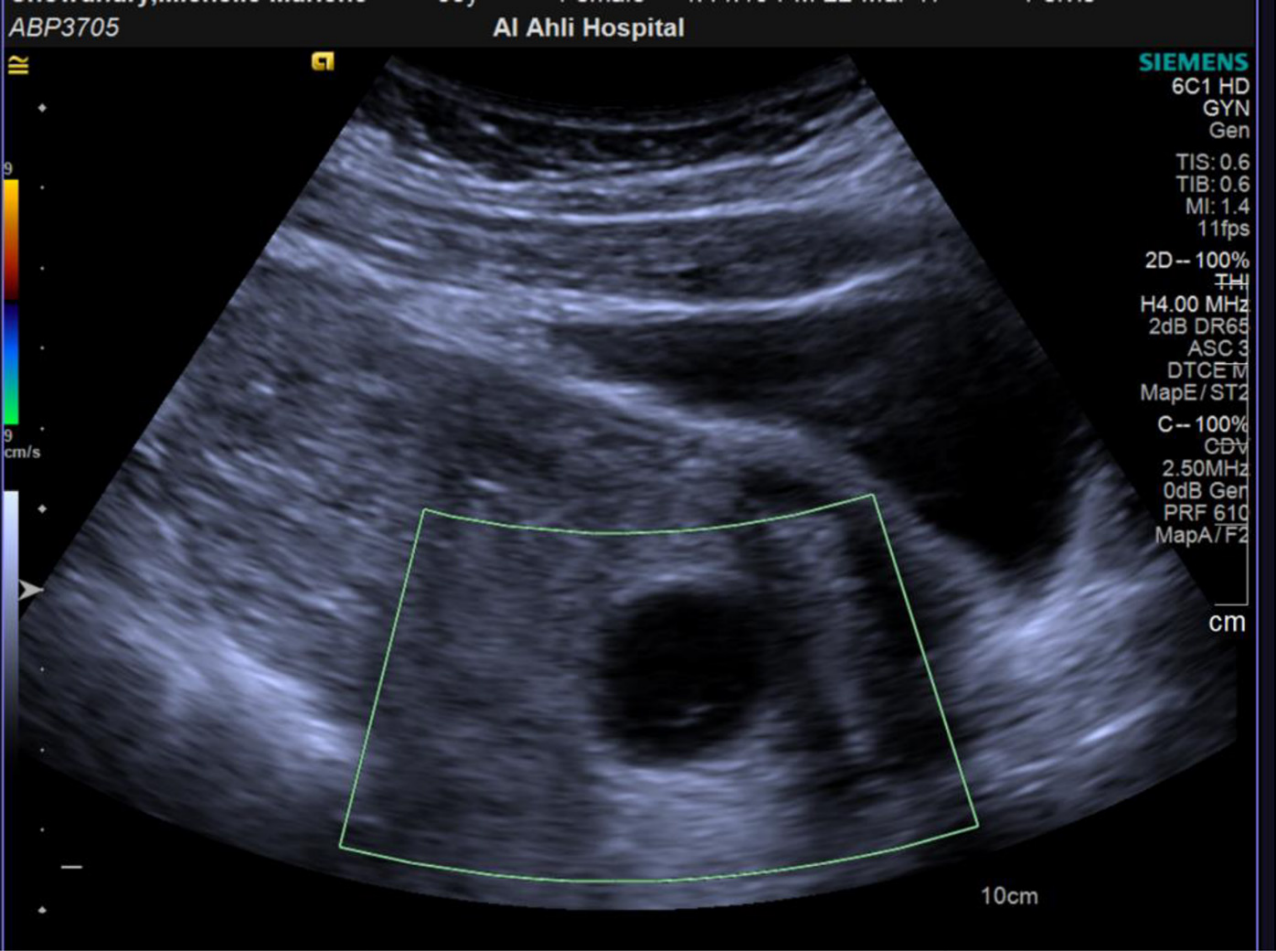
Absent vascularity on color Doppler.

**Figure 2.**
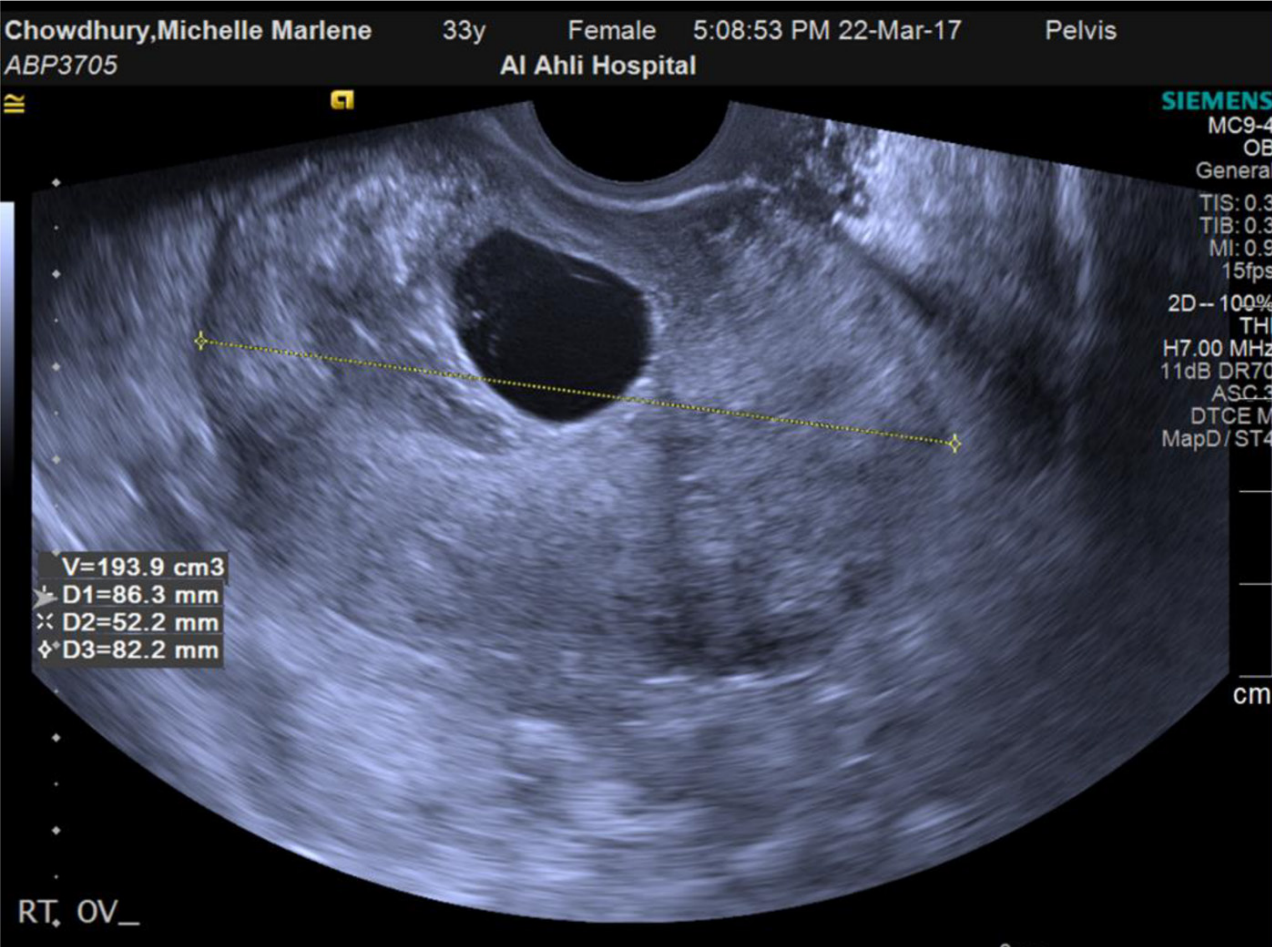
Enlarged ovary and edematous stroma with corpus luteum cyst.

Laboratory findings included BHCG: 33931 mIU/ml, Hb: 12.4g/dl, WBCs: 15.78 (10e3/uL), CRP: <5 mg/l. On ultrasound examination, an intrauterine viable pregnancy at six weeks was detected. The right ovary was enlarged with a size of 8.6×5.2×8.2 cm. It was edematous with no vascularity (highly suspicious of ovarian torsion), as shown in Figures 1 and 2.

Emergency operative laparoscopy under general anesthesia was decided. The procedure was a right salpingo-oophorectomy due to a gangrenous ovary. Tissues were sent for histopathology, which showed a right tube-ovarian torsion with a hypertrophic corpus luteum, and all tissues were infarcted and necrotic.

The patient was started on progesterone vaginal pessaries 100 mg tds (Endometrin, Ferring), progesterone oral tablet 20 mg bid (Duphaston, Abbot),17 hydroxyprogesterone caproate 500mg IM twice weekly (Proluton depot, Bayer) and folic acid. The treatment was given according to the hospital protocol for bleeding cases in early pregnancy (based on NICE guidelines).^17^ She did not have vaginal bleeding after the surgery. She was discharged after seven days while ordered to continue the same medications.

The patient had regular weekly follow up in the clinic, complained of pelvic pain episodes, and was admitted with vaginal bleeding at 12 weeks. In every visit, an ultrasound was done to confirm the fetus’s viability and assess the pregnancy’s progress. Endometrin suppositories were replaced by progesterone rectal suppository 400 mg tds (Cyclogest, LD Collins) at 12 weeks (the Endometrin was changed because the patient had vaginal irritation).

She was re-admitted to the hospital in the 21^st^ week of pregnancy with severe pelvic heaviness and pain. Ultrasound showed an incompetent short cervix of 2 cm in length with funneling (gap of 9.8 mm at the internal os). Therefore, it was decided to do an emergency McDonald’s cerclage operation.

The patient continued the same medications until 20 weeks. After that, the dose was reduced to Duphaston 20 mg bid, Cyclogest 400 mg bid, and Proluton depot 500mg once weekly for two weeks. Then she received only Proluton depot 250 mg once weekly and Cyclogest 400 mg bid until 29 weeks. Her follow-up visits were uneventful until 29 weeks; then, she decided to travel to the United States of America for delivery; she reached her 35th week and delivered a healthy baby boy.

## Discussions

Ovarian stimulation is a known risk factor for ovarian torsion. Ovarian torsion should always be suspected in patients undergoing infertility interventions with severe abdominal pain. The case could be misdiagnosed as other diseases that cause lower abdominal pain, such as acute appendicitis, renal colic, and ectopic pregnancy. The diagnosis was not missed in our case due to a strong history of infertility treatment, clinical signs, and ultrasound examination.

It was not easy to preserve the ovary since the tissues remained gangrenous after the detorsion of the adnexa that was attempted to re-vascularize the ovary. We had to do a salpingo-oophorectomy. The histopathology report confirmed that the ovary and the tube were gangrenous and necrotic.

Our case of adnexal torsion was managed by an emergency operative laparoscopy for right salpingo-oophorectomy under general anesthesia. Laparoscopy is considered safe in pregnancy. Studies comparing laparoscopy with laparotomy demonstrated improved visualizing of pelvic organs, less blood loss, and reduced risk of uterine irritability in laparoscopic surgeries.^17^ The two procedures have no difference in intrauterine fetal growth restriction or stillbirth rates.^18^ Laparoscopic techniques do not seem to modify uteroplacental perfusion.^19^

In our case, as removing the affected ovary, including the corpus luteum cyst, was compulsory, it was mandatory to start progesterone as a replacement to allow the continuation of pregnancy. That was supported by C sapo et al. 1972, who concluded that patients who have been submitted to oophorectomy at about seven weeks of pregnancy would have a decreased progesterone level and abort.^3,20^

The progesterone treatment was planned according to Al-Ahli Hospital protocol, but the dose was increased based on our clinical assessment. We continued progesterone supplementation to our patient until the end of her follow-up at 29 weeks, but the dose was reduced after 20 weeks.

The National Institute for Health & Excellence (NICE) guideline in 2021 recommends using vaginal micronized progesterone 400 mg twice daily up to 16 weeks into pregnancy in women with bleeding at early pregnancy and with previous miscarriage. The recommendations were based on the PRISM trial and other systematic reviews.^21^

Coomarasamy et al. 2019,^22^ in the PRISM trial (Progesterone to prevent miscarriage in women with early pregnancy vaginal bleeding), have chosen the treatment window until 16 weeks based on the rationale that progesterone production from the corpus luteum after 16 weeks of pregnancy becomes less critical when compared to the placental production of progesterone after 16 weeks. Both corpus luteal removal and vaginal bleeding in early pregnancy are associated with progesterone deficiency and can be treated similarly.

17 hydroxyprogesterone caproate is used to prevent preterm labor.^23^ The Food and Drug Administration (FDA) had approved its use during pregnancy to reduce the risk of preterm delivery before 34 weeks.^24^ We advised the patient to continue progesterone supplementation until the 34th week of gestation.

The NICE guideline recommends using vaginal progesterone up to 34 weeks for women with a history of spontaneous preterm birth or short cervix.^25^ Deng et al. 2020 found a greater risk of abortion when the progesterone level was less than 90.62 mmol/L.^26^ The findings of this association between the serum progesterone level and the risk of miscarriage can better assist the clinician in understanding patients’ conditions and making decisions. Studies showed that low progesterone was associated with abortion.^27^

In our case, serial measurements of progesterone levels were not done. The pregnancy was progressing generally with progesterone supplementation. Serum progesterone values may be considered a reference for progesterone supplementation, but further studies are needed to verify whether serum progesterone is a better indication for progesterone replacement.^22^

Our case was admitted at 21 weeks for an ultrasound-based cervical cerclage. Cervical cerclage is known to be one of the standard options for prophylactic intervention in the care of women at risk of preterm birth and second-trimester fetal loss. In women with singleton pregnancy, insertion of an emergency cerclage may delay birth by an average of 34 days, compared with expected management in suitable cases. It may be associated with a two folds reduction in the chance of delivery before 34 weeks of gestation.^28^

## Conclusions

Ovarian torsion in pregnancy is a severe gynecological emergency. Removing the corpus luteum that supports early pregnancy requires hormonal treatment in higher doses than recommended for threatened abortion.

## References

[1] Feng J-L, Zheng J, Lei T, Xu Y-J, Pang H, Xie H-N (2020;). Comparison of ovarian torsion between pregnant and non-pregnant women at reproductive ages: Sonographic and pathologic findings. Quant Imaging Med Surg.

[2] Young R, Cork K (2017;). Intermittent ovarian torsion in pregnancy. Clin Pract Cases Emerg Med.

[3] Duncan WC (2021;). The inadequate corpus luteum. Reprod Fertil.

[4] Hibbard LT (1985;). Adnexal torsion. Am J Obstet Gynecol.

[5] Bignardi T, Condous G (2009;). The management of ovarian pathology in pregnancy. Best Pract Res Clin Obstet Gynaecol.

[6] Gorkemli H, Camus M, Clasen K (2002;). Adnexal torsion after gonadotrophin ovulation induction for IVF or ICSI and its conservative treatment. Arch Gynecol Obstet.

[7] Pride SM, James CSJ, Yuen BH (1990;). The ovarian hyperstimulation syndrome. Semin Reprod Endocrinol.

[8] American Society of reproductive medicine (2008;). Ovarian hyperstimulation syndrome {OHSS}. Fertil Steril.

[9] Huchon C, Fauconnier A (2010;). Adnexal torsion: A literature review. Eur J Obstet Gynecol Reprod Biol.

[10] Glanc P, Salem S, Farine D (2008;). Adnexal masses in the pregnant patient: A diagnostic and management challenge. Ultrasound Q.

[11] Hess LW, Peaceman A, O’Brien WF, Winkle CA, Cruishank DP, Morrison JC (1988;). Adnexal mass occurring with intrauterine pregnancy: Report of 54 patients requiring laparotomy for definitive management. Am J Obstet Gynecol.

[12] Charlie C (2021). Adnexal torsion. Baylor College of Medicine.

[13] Shadinger LL, Andreotti RF, Kurian RL (2008;). Preoperative sonographic and clinical characteristics as predictors of ovarian torsion. J Ultrasound Med.

[14] El-Shawarby SA, Henderson AF, Mossa MA (2005;). Ovarian cysts during pregnancy: Dilemmas in diagnosis and management. J Obstet Gynecol.

[15] Shur J, Bottomley C, Walton K, Patel JH (2018;). Imaging of acute abdominal pain in the third trimester of pregnancy. BMJ.

[16] Shah D, Nagarajan N (2013;). Luteal insufficiency in the first trimester. Indian J Endocrinol Metab.

[17] Chen L, Ding J, Hua K (2014;). Comparative analysis of laparoscopy versus laparotomy in managing ovarian cyst during pregnancy. J Obstet Gynecol Res.

[18] Reedy MB, Kallén B, Kuehl TJ (1997;). Laparoscopy during pregnancy: A study of five fetal outcome parameters using the Swedish Health Registry. Am J Obstet Gynecol.

[19] Candiani M, Maddalena S, Barbieri M, Izzo S, Alberico D, Ronzoni S (2012;). Adnexal masses in pregnancy: Fetomaternal blood flow indices during laparoscopic surgery. J Minim Invasive Gynecol.

[20] Csapo AI, Pulkkinen MO, Ruttner B, Sauvage JP, Wiest WG (1972;). The significance of the human corpus luteum in pregnancy maintenance. I. Preliminary studies. Am J Obstet Gynecol.

[21] National Institute for Health and Clinical Excellence Ectopic pregnancy and miscarriage: Diagnosis and initial management. NICE guideline [NG 126] /2019.

[22] Coomarasamy A, Harb HM, Devall AJ, Cheed V, Roberts TE, Goranitis I, et al (2020;). Progesterone to prevent miscarriage in women with early pregnancy bleeding: The PRISM RCT. Health Technol Assess.

[23] Norwitz ER, Caughey AB (2011;). Progesterone Supplementation and the Prevention of Preterm Birth. Rev Obstet Gynecol.

[24] Food and Drug Administration (FDA) Application number: 21945Orig1s000.

[25] National Institute for Health and Clinical Excellence Preterm labor and birth. NICE guideline [NG 25] /2022.

[26] Deng Y, Chen C, Luo S, Mai G, Liao X, Tian H (2020;). Baseline serum progesterone levels and the first-trimester pregnancy outcome in women with threatened abortion: A retrospective cohort study. Biomed Res Int.

[27] Siew JYS, Allen JC, Hui CYY, Ku CW, Malhorta R, Ostbye T (2018;). The randomized controlled trial of micronized progesterone and dydrogesterone (TRo MaD) for threatened miscarriage. Eur J Obstet Gynecol Reprod Biol.

[28] Royal College of Obstetricians and Gynaecologists (RCOG) (2022). Cervical cerclage: Green top guideline No 75.

